# Amygdala activation to threat under attentional load in individuals with anxiety disorder

**DOI:** 10.1186/2045-5380-1-12

**Published:** 2011-12-16

**Authors:** Thomas Straube, Judith Lipka, Andreas Sauer, Martin Mothes-Lasch, Wolfgang HR Miltner

**Affiliations:** 1 Department of Biological and Clinical Psychology, Friedrich-Schiller-University, Jena, Germany

## Abstract

**Background:**

Previous studies in healthy subjects have shown that strong attentional distraction prevents the amygdala from responding to threat stimuli. Here, we investigated the effects of attentional load on amygdala activation to threat-related stimuli in individuals suffering from an anxiety disorder.

**Methods:**

During functional magnetic resonance imaging, spider-phobicand healthy control subjects were presented with phobia-related and neutral stimuli while performing a distraction task with varying perceptual load (high vs low).

**Results:**

Our data revealed a pattern of simultaneously increased amygdala and visual cortical activation to threat vs neutral pictures in phobic individuals, compared with controls, occurring regardless of attentional load.

**Conclusions:**

These results suggest that, in contrast to studies in healthy subjects, amygdala activation to clinically relevant threat stimuli is more resistant to attentional load.

## Background

In accordance with theories suggesting a critical function of the amygdala in the processing of threat signals and the mediation of fear responses [1,2], several studies found increased amygdala activation to threatening vs neutral stimuli in individuals with anxiety disorders [3-8] and in healthy subjects [9-14]. Furthermore, there are strong theoretical accounts proposing an automatic response of the amygdala to threat signals even when target stimuli are presented during attentional distraction [1,2,14]. Whereas some studies indeed suggest an automaticity of amygdala activation to threat-related stimuli under conditions of attentional distraction [9,13,14], several recent studies in healthy subjects, however, indicated a complete inhibition of differential activation to threat vs neutral stimuli within the amygdala, given sufficiently strong perceptual load by a main task [15-18]. Thus, it seems that, at least in healthy subjects, automatic activation of the amygdala to emotional stimuli does not occur when demanding cognitive tasks exhaust the available processing resources.

Bishop *et al.*, for example, used a perceptual load task, while subjects were exposed to fearful and neutral faces. Perceptual load was induced by varying the number of task-relevant items [19,20] within a letter string presented along with the facial expression. When perceptual identification was easy (low load), elevated state anxiety was associated with a heightened response to threat distractors in the amygdala and superior temporal sulcus, whereas individuals scoring high in trait anxiety showed a reduced prefrontal response to these stimuli. The latter finding was interpreted as weakened recruitment of control mechanisms when confronted with salient distractors. This finding is in accordance with theories assuming an imbalance between the stimulus-driven processing of salient threat-related stimuli, associated with automatic orienting, and goal-directed attentional control (for example, [21]). This would lead to a relatively stronger role of the posterior attentional systems in the brain involved in bottom-up attention as compared to the more anterior top-down control system [22]. However, in the study of Bishop *et al.*, neither high-anxious nor low-anxious subjects showed an increased amygdala response to threat distractors when perceptual identification was more attention demanding (high perceptual load). Furthermore, high attentional load in previous studies also prevented differential activation to threat vs neutral stimuli in areas of the extrastriate visual cortex, suggesting the absence of differential processing of threat and neutral stimuli also in areas beyond the amygdala [15,17,23].

Thus, in line with a recent model of selective attention [19,20], processing of task-unrelated stimuli is prevented when task-related demands exhaust perceptual capacity limits. Even though this model does not predict that also the processing of salient emotional stimuli is impaired by high perceptual load [24], it has been extended to the domain of threat processing [15,23]. Furthermore, based on the findings of Bishop *et al.*, effects of subjects' anxiety on the neural processing of threat-related stimuli seem to appear only during relatively low-load tasks. Thus, high load should prevent neural responses to threat and also the attentional processing of these stimuli, that is automatic orienting [22]. This position is in contrast to cognitive models of anxiety [25] predicting a mandatory processing of threat stimuli in anxious subjects or models predicting that anxiety increases the processing of threat-related signals under high demands on the central executive [21].

Even though it has been shown that high perceptual load prevents the processing of threat stimuli in anxious healthy subjects, it is unknown whether similar findings will be observed in individuals suffering from an anxiety disorder. Automatic processing of disorder-related stimuli seems to be a main feature of anxiety disorders and this might be represented in attention-independent activation of the amygdala [1,2,8]. An example is specific phobia, which is among the most common anxiety disorders [26]. Neuroimaging research implicates the amygdala in the processing of phobia-related stimuli, specifically in the initial detection of such stimuli and perhaps in the lowering of thresholds for the induction of rapid fear responses, rather than in the sustained processing of phobia-relevant information [8,27,28]. For example, activation of the amygdala in spider-phobic subjects has been demonstrated regardless of whether attention was focused on the stimuli or distracted by an unrelated foreground task [8], supporting the hypothesis that the amygdala is automatically activated by phobogenic stimuli [1,2,29]. Furthermore, this attention independent response in the amygdala was associated with increased activation in the extrastriate visual cortex [8], which is typically coactivated with the amygdala in spider phobia in response to phobia-related stimuli (for example, [3,30-32]).

A recent study with spider-phobic subjects reported attention-dependent activation of the amygdala to spider pictures [33]. However, in this study the number of phobia-related stimuli and attention focus were confounded, making a clear interpretation of the results difficult. Thus, the findings might even be interpreted to support the hypothesis of automatic amygdala activation to task-irrelevant (background) spider pictures. To date, there has been no functional imaging study that employed a parametric variation of attentional load in individuals with specific phobia or any other anxiety disorder.

In the present study, we used event-related functional magnetic resonance imaging (fMRI) to explore the question whether amygdala activation to phobia-relevant stimuli is modulated by a parametric variation of attentional distraction in patients with specific phobia. We used a perceptual load task that has been previously shown to inhibit amygdala activation to threat-related stimuli in high-anxious healthy subjects [15]. An absence of attentional modulation of amygdala activation in the present experiment would indicate a role of the amygdala in threat processing even under high attentional load in individuals with anxiety disorder. Additionally, we examined the neural activation in the visual cortex and several brain areas proposed to be involved in the processing of threat-related stimuli.

The results show a pattern of simultaneously increased amygdala and visual cortical activation to threat vs neutral pictures in phobic individuals, compared with controls, occurring regardless of attentional load. These findings suggest that amygdala activation to clinically relevant threat stimuli is resistant to attentional load.

## Methods

### Subjects

A total of 17 spider-phobic (mean age = 25.2, SD = 4.9) and 16 control subjects (mean age = 26.6, SD = 9.2) participated in the study. Participants were right handed female university students with normal or corrected-to-normal vision who provided written informed consent to volunteer in the study. The ethics committee of the University of Jena approved all experimental procedures. All phobic subjects fulfilled the diagnostic criteria for spider phobia according to the *Diagnostic and Statistical Manual of Mental Disorders*, fourth edition (DSM-IV; [34]) as assessed by a structured clinical interview [35]. According to this interview, spider-phobic subjects had no additional psychopathological disorders. In addition, spider-phobic subjects, but not controls, showed high scores on a spider phobia questionnaire ([36]; mean = 23.4, SD = 2.3 vs mean = 2.8, SD = 1.6; t = 29.55, *P *< 0.001). There was no difference in trait or state anxiety scores between groups ([37]; trait: mean phobics = 36.94, SD = 11.02, mean controls = 41.93, SD = 7.40; t = 1.43, *P *> 0.05; state: mean phobics = 38.27, SD = 10.42, mean controls = 34.45, SD = 4.45; t = 1.27, *P *> 0.05). Further demographic and clinical characteristics are summarized in Table 1.

**Table 1:** Demographic and clinical characteristics

	Phobic subjects (N = 17)	Healthy controls (N = 16)
Age in years, mean (SD)	25.2 (4.9)	26.6 (9.2)
Ethnicity	Caucasian	Caucasian
Education	At least secondary high school	At least secondary high school
Prior/current medication	No	No
Psychotherapy	No	No
SPQ, mean (SD)	23.4 (2.3)	2.8 (1.6)
STAI, mean (SD)	36.94 (11.02)	34.45 (4.45)

SPQ = spider phobia questionnaire; STAI = State-Trait Anxiety Index.

### Stimuli and tasks

**Figure 1:**
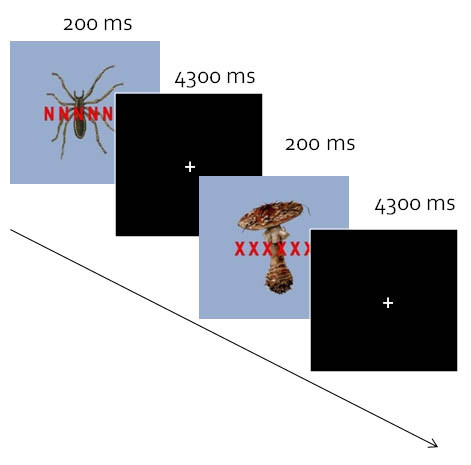
**Example of the design**. Shown are two trials of a low load mini block. Each load block consisted of four different randomly selected pictures. Load was varied across blocks and picture category was varied within blocks.

Subjects were exposed to 48 different pictures of spiders and 48 different pictures of mushrooms while performing a letter search task (adapted from [15]). The spider pictures represented the disorder-related stimuli. We used mushrooms as control stimuli, mainly for reasons of comparability with several previous studies (for example, [8,12,38,39]). A string of six letters written in red ink was superimposed onto the task-irrelevant spider or mushroom picture, respectively. In half of the trials (high perceptual load), the string comprised a single target letter (N or X) and five non-target letters (H, K, M, W, Z), which were arranged in random order. In the other half of the trials (low load), the letter string comprised either six Xs or six Ns. The task was to decide by button press whether the letter string contained an 'X' or an 'N'. The low-load and high-load conditions were arranged in blocks of four trials (see [15]). In total, there were 24 blocks of 4 trials each. Load was varied across blocks and picture category was varied within blocks. The stimulus onset asynchrony (SOA) was 4.5 sec allowing improved sampling of the BOLD response due to jittering between SOA relative to the repetition time (TR), thus representing an effective interval for event-related designs. The pictures were randomized across and within blocks with the restriction that two mushroom and two spider pictures were shown within each block. The stimuli (including the letter strings) were presented for 200 ms in random order with a resulting interstimulus interval of 4300 ms. Figure 1 shows an example of two trials. The overall picture size was 15 × 20° visual angle, with the stimuli subtending approximately 11.5 × 11.5°. After the fMRI session, participants rated the pictures using a nine-point Likert scale to assess valence (1 = 'very pleasant' to 9 = 'very unpleasant') and arousal (1 = 'not arousing' to 9 = 'very arousing'). Behavioral data were analyzed by repeated measures analysis of variance using SPSS (V. 17; SPSS, Chicago, IL, USA) with subsequent post hoc t tests (Bonferroni corrected). For analysis of performance data, one control subject had to be excluded due to technical problems during the registration of button presses.

### fMRI data acquisition and analysis

A run of 294 volumes (40 axial slices per volume, thickness = 3 mm, in plane resolution = 3 × 3 mm) was acquired (3 T; 'Tim Trio', Siemens, Erlangen, Germany) using a T2*-weighted echo planar sequence (echo time (TE) = 30 ms, flip angle = 90°, matrix = 64 × 64, field of view (FOV) = 192 mm, TR = 2.9 s). Additionally, a T1-weighted anatomical volume was recorded (192 slices, echo time (TE) = 6 ms, matrix = 256 × 256, voxel size = 1 × 1 × 1 mm). Preprocessing and analysis of the functional data were performed using the software Brain Voyager QX (Brain Innovation, Maastricht, The Netherlands). All volumes were realigned to the first, corrected for slice time errors, and spatially (8 mm full-width half-maximum isotropic Gaussian kernel) as well as temporally (high pass filter: cut-off = 0.006 Hz) smoothed. Furthermore, data preprocessing included removal of linear trends and of the mean. Anatomical and functional images were coregistered and normalized to the Talairach space. Statistical analysis was performed by multiple linear regression of the signal time course at each voxel. The expected blood oxygen level-dependent signal change for each predictor was modeled by a hemodynamic response function (based on a two-gamma-function model, which models rise and undershoot of the BOLD response, as implemented in Brain Voyager). Predictors of non-interest were the six movement parameters. The four predictors of interest were the spider pictures/low load, spider pictures/high load, mushroom pictures/low load, and mushroom pictures/high load. Statistical comparisons were conducted using a mixed-effect analysis. In the first step, voxelwise statistical maps were generated and predictor estimates (β weights) were computed for each individual. In the second step, contrasts of predictor estimates were analyzed across subjects with repeated-measures analysis of variance (ANOVA). Statistical parametric maps resulting from the voxelwise analysis were considered significant for statistical values that survived a cluster-based correction for multiple comparisons. Voxel-level threshold was initially set to *P <*0.005 (uncorrected) to strike a balance between type I and type II errors. Thresholded maps were then submitted to a region of interest (ROI)-specific or whole brain-specific correction criterion, which was based on the estimate of the map's spatial smoothness and on an iterative procedure (Monte Carlo simulation) used to estimate cluster-level false-positive rates. After 1,000 iterations, the minimum cluster size threshold that yielded a cluster-level false-positive rate of 5% was applied to the statistical maps (11 voxels for whole brain analysis). According to our previous studies [7,8,12,13,18,32], the following anatomical ROIs were defined a priori using Talairach daemon software [40,41]: amygdala, insula, anterior cingulate cortex (ACC), dorsolateral prefrontal cortex (DLPFC), dorsomedial prefrontal cortex (DMPFC), and fusiform gyrus, with the latter region consistently shown to be involved in the visual processing of spider pictures in spider-phobic subjects (for example, [8]). Statistical data are only shown for significantly activated voxels.

## Results

### Performance data

For accuracy (Table 2), a main effect of load was found (F(1,30) = 70.8, *P <*0.0001) due to decreased accuracy during the high-load condition. For reaction times (Table 2), a main effect of load (F(1,30) = 201.6, *P <*0.0001), due to increased reaction times during the high-load condition, and an interaction of Task × picture category (F(1,30) = 6.5, *P <*0.05), due to increased reaction times to spiders vs mushrooms during low but not high load, were found.

**Table 2:** Behavioral data

	Phobics		Controls	
	
	Spider	Mushroom	Spider	Mushroom
Performance				
Reaction times (ms):				
High load	973.04 (166.0)	986.8 (160.98)	1047.51 (154.37)	1062.14 (152.33)
Low load	683.79 (146.9)	664.4 (108.6)	665.79 (58.75)	655.85 (61.54)
Accuracy (% correct):				
High load	68.38 (12.48)	62.38 (17.16)	67.5 (11.16)	64.31 (16.31)
Low load	87.87 (16.09)	87.13 (18.48)	88.01 (22.08)	90.0 (22.27)
Ratings				
Valence (range 1 to 9)	8.59 (0.44)	3.41 (1.79)	5.46 (1.06)	4.58 (1.05)
Arousal (range 1 to 9)	8.24 (0.69)	1.05 (0.30)	2.89 (1.87)	1.08 (0.36)

Data shown are mean (SD).

### Rating data

Post-scanning arousal and valence ratings (Table 2) showed a main effect of group (F(1,30) = 101.3, *P <*0.0001; F(1,30) = 11.3, *P <*0.005), picture category (F(1,30) = 324.9, F(1,30) = 102.4, both *P <*0.0001), and an interaction of group × picture category (F(1,30) = 116.0, F(1,30) = 51.6, both *P <*0.0001). Post hoc analysis using t tests (Bonferroni corrected) revealed that phobic subjects rated spiders, but not mushrooms, as more arousing and unpleasant than control subjects (arousal: t = 10.7, *P <*0.001 (spiders), t = 0.3; *P >*0.05 (mushrooms); t = 10.9, *P <*0.001 (spiders), t = -2.0; *P >*0.05 (mushrooms)).

### fMRI analysis

#### Amygdala ROI

**Figure 2:**
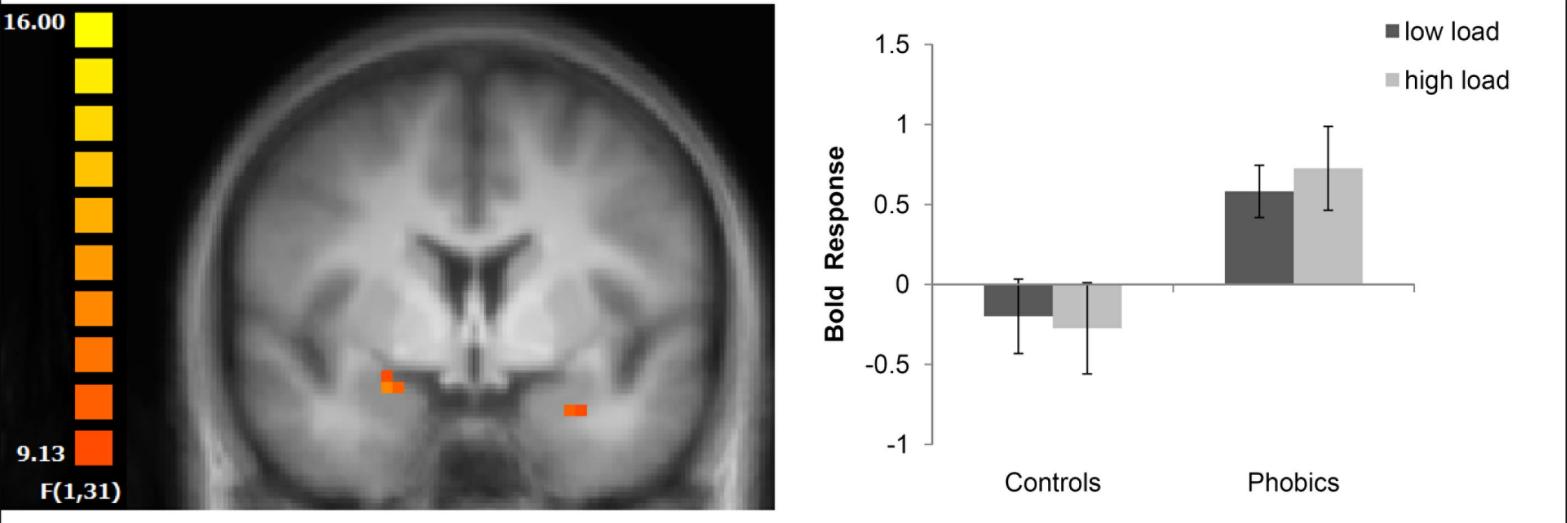
**Amygdala responses to spider vs mushroom pictures**. Increased activation in the right and left amygdala in phobic subjects was found regardless of perceptual load. Statistical parametric maps are overlaid on a T1 scan (radiological convention: left = right; y = -1). The plot shows the difference of parameter estimates (spider vs mushroom; mean and standard error) for the maximally activated voxel in the left amygdala.

For both the right and left amygdala, there was only a main effect of load (left: (x, y, z) = -25, -8, -12; *F*[1,31] = 45.09; right: (x, y, z) = 27, -10, -11; *F*[1,31] = 55.54; both *P *< 0.05, corrected; cluster size: left: 2771 mm^3^; right: 2571 mm^3^) and an interaction of group × picture category (left: (x, y, z) = -27, -1, -17; *F*[1,31] = 10.15; right: (x, y, z) = 23, -1, -11; *F*[1,31] = 11.54; both *P *< 0.05, corrected; cluster size: left: 108 mm^3^; right: 116 mm^3^). Thus, our data did not reveal an interaction of group × picture category × load. The main effect of load resulted from decreased amygdala activation across pictures and groups under high vs low load. The interaction of group by picture category was due to increased activation to spider versus neutral pictures in phobic subjects, as compared to healthy controls. However, as also indicated in Figure 2, the increased activation to threat vs neutral pictures in phobic subjects was independent of perceptual load.

#### Other ROIs and whole brain analysis

As indicated in Table 3, an effect of load was also evident in several other brain regions. While regions such as the dorsal ACC and other areas in the frontal and parietal cortex implicated in dealing with task difficulty showed increased activation during high load, other areas such as the ventromedial prefrontal cortex, which is typically deactivated during demanding tasks, as well as areas in the visual cortex showed a decreased activation under high vs low load.

**Table 3:** Significant brain activation

Area	Side	x	y	z	Size (mm^3^)	F value	Signal change (%)
Main effect load (ROI):							
Amygdala	R	27	-10	-11	2571	55.54	0.35
	L	-25	-8	-12	2771	45.09	0.35
Insula	R	33	15	11	1674	54.72	0.43
	L	-35	15	12	1431	59.68	0.43
FG	L	-45	-55	-14	662	45.06	0.49
Dorsal ACC	R/L	-7	43	11	10652	65.54	0.70
DMPFC	R/L	-10	43	15	8391	54.61	0.45
DLPFC	R	23	23	52	1489	42.11	0.38
	L	-19	28	52	3604	57.51	0.53
Main effect load (whole brain):							
Parietal cortex	R	2	-53	21	11751	62.70	0.76
	L	-25	-66	34	17118	103.44	0.83
VMPFC	R/L	-7	43	9	4503	71.67	0.70
Visual cortex	R	3	-77	-7	373	23.06	0.37
	L	-3	-81	-8	328	27.06	0.36
Main effect picture (whole brain):							
STG	R	53	-14	-3	377	13.12	0.14
Visual cortex	R	16	-93	-5	783	20.79	0.19
	L	-18	-94	-10	513	22.57	0.21
Interaction picture by group (ROI):							
Amygdala	R	23	-1	-11	116	11.54	0.40
	L	-27	-1	-17	108	10.15	0.35
FG	L	-42	-55	-11	179	12.11	0.32

x, y, z are the Talairach coordinates of peak voxel activation threshold: *P *< 0.05, corrected. Cluster threshold whole brain: 11 voxels; other thresholds: 3 to 5 voxels; voxel threshold: *P *< 0.005.

ACC = anterior cingulate cortex; DLPFC = dorsolateral prefrontal cortex; DMPFC = dorsomedial prefrontal cortex; FG = fusiform gyrus; ROI = region of interest; STG = superior temporal gyrus; VMPFC = ventromedial prefrontal cortex.

**Figure 3:**
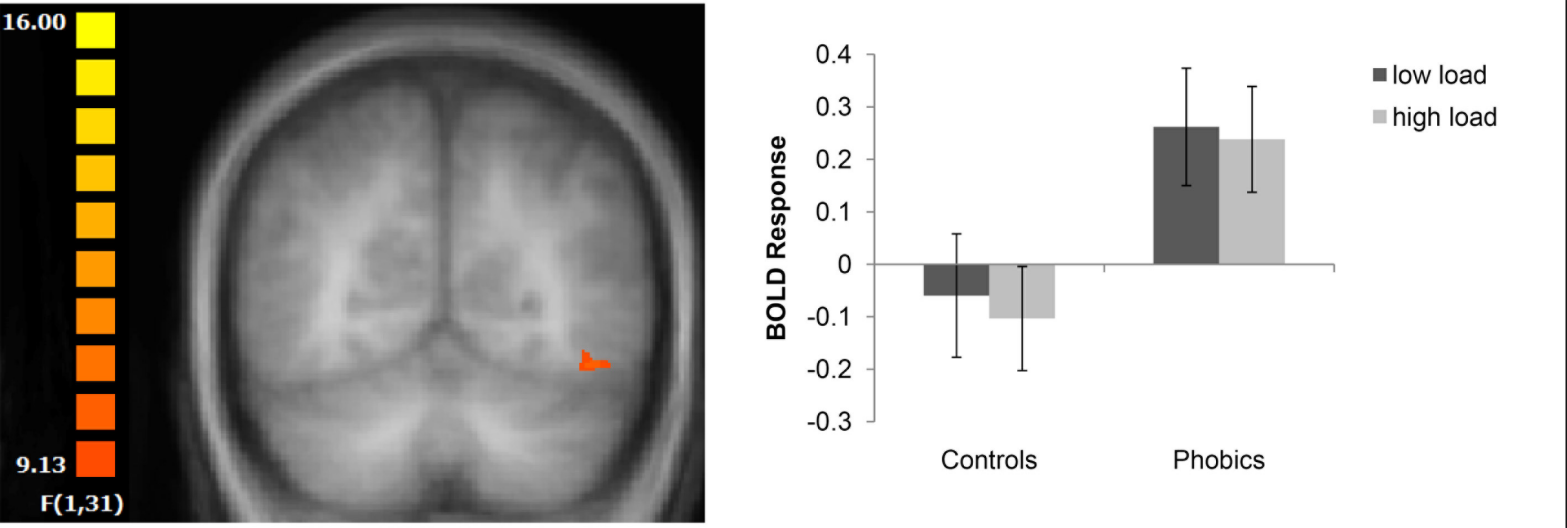
**Activation to spider vs mushroom pictures in the extrastriate visual cortex**. Increased activation in the left fusiform gyrus in phobic subjects was found regardless of perceptual load. Statistical parametric maps are overlaid on a T1 scan (radiological convention: left = right; y = -55). The plot shows the difference of parameter estimates (spider vs mushroom; mean and standard error) for the maximally activated voxel.

Furthermore, there were main effects of picture category in the superior temporal gyrus and the visual cortex due to decreased (superior temporal gyrus (STG)) and increased (visual cortex) activation to spiders vs mushrooms across subjects and tasks (see Table 3). Most importantly, there was an interaction of group × picture specifically in the left fusiform gyrus (see Table 2 and Figure 2). The interaction of group by picture category was due to increased activation to spiders versus neutral pictures in phobic subjects, as compared to healthy controls. As also indicated in Figure 3, the increased activation to threat vs neutral pictures in phobic subjects was independent of perceptual load, comparable with the profile of activation in the amygdala. There were no further significant main effects or interactions.

## Discussion

The present study provides evidence for a critical involvement of the amygdala in threat processing under attentional load in subjects suffering from an anxiety disorder. Thus, amygdala activation to disorder-related vs neutral stimuli was observed regardless of attentional load. A similar finding was evident for the left fusiform gyrus.

This finding contrasts with prior studies in healthy subjects [15-18], especially with a study where the same task resulted in a strong modulation of amygdala activation to fearful vs neutral faces, with the differential amygdala activation completely inhibited under the high-load condition [15]. The study of Bishop *et al. *found that, during a low-load condition, when perceptual distractor identification was less demanding, elevated state anxiety was associated with a heightened response to fearful faces in the amygdala and superior temporal sulcus, whereas individuals scoring high in trait anxiety showed a reduced prefrontal response to these stimuli. The latter finding was interpreted to indicate a weakened recruitment of cortical control mechanisms in anxious individuals when confronted with threat distractors. However, neither high-anxious nor low-anxious volunteers showed an increased amygdala response to threat distractors when the perceptual identification task was more attention demanding (high perceptual load).

Thus, it seems that the findings in subjects showing high, but subclinical, levels of state and trait anxiety may not necessarily be comparable to those of a subject sample meeting the diagnostic criteria for a clinically relevant anxiety disorder such as specific phobia. This difference suggests an increased responsiveness of the amygdala to threat signals in anxiety disorder patients. This increased responsiveness might be mainly associated with differences in the threat relevance of stimuli used in the different studies. Thus, while fearful facial expressions are associated with rather low anxiety ratings, disorder-related stimuli evoke strong fear responses in subjects suffering from an anxiety disorder. Furthermore, our findings are also not associated with trait or state anxiety scores of subjects, since there was no difference between groups. Thus, the use of increased trait or state scores as analogue to clinical disorders might be often of limited relevance. Rather, it may be the disorder-related importance of the stimuli that determines differential brain responses, at least in phobias.

Our results support previous findings of amygdala activation to threat under conditions of attentional distraction in specific phobia [8,33]. However, no previous study in individuals with anxiety disorders employed a parametric manipulation of attentional load as yet. Also, prior work may differ in that the distraction conditions might not have been very demanding [8], or that they were confounded with other factors [33]. The present results suggest that, at least in specific phobia, the salience of stimuli evokes differential amygdala activation to threat vs neutral stimuli independent of attentional load, even though the amygdala and other areas were found to be modulated by attentional load in general. Thus, high load led to decreased activation of the amygdala and several other brain areas. Conversely, regions implicated in attentional control and dealing with task difficulty showed increased activation under high as compared to low load. This general effect of load or attentional distraction is in line with prior work [15,17,23].

We did not detect any evidence for a decreased prefrontal control of threat distractors as suggested by Bishop *et al. *However, one has to keep in mind that the results in the Bishop *et al. *study are based on a correlation with trait anxiety scores and a comparable significance of the facial expressions for all subjects (high and low anxious). Here, we compared subjects with anxiety disorder to healthy controls. That is, for spider-phobic subjects the spider pictures were disorder related, while for the control group the (attentional control of these) pictures had no relevance. This prevents a meaningful comparison of differential control mechanisms between groups.

Beyond its role in the rapid induction of defense behaviors [1,2], the amygdala might also be involved in attentional functions [2,42-45], for example, through the modulation of activation in visual areas by feedback connections [46]. This influence of the amygdala might allow the enhanced perception of threat [47]. Accordingly, it has been shown that the amygdala drives the activation of areas within the inferior temporo-occipital cortex such as the fusiform gyrus [14] and increased activations to threat even under distraction conditions or perceptual unawareness have been found in visual areas [48,49]. In line with these findings, our data revealed a significant activation of the fusiform gyrus to spider vs neutral stimuli in spider-phobic subjects occurring in conjunction with the amygdala activation during both attentional conditions.

The amygdala's influence on attentional functions is not specific for anxious subjects or anxiety disorders, but can be found in healthy subjects as well (for example, [43,44,50]). Animal research also implicates the amygdala in forming a crucial part of a pervasive vigilance system subserving facilitated processing of biologically relevant information [45,51,52]. Thus, the meaning of automatic amygdala activations for phobic symptomatology might be associated with such functions. Individuals suffering from specific phobias show increased vigilance for phobia-relevant stimuli [48,53]. Under divided attention conditions, the amygdala might be activated even by crude representations of threat stimuli requiring the brain to gather more information by potentiating subsequent sensory information processing.

It should be noted that we do not suggest that these findings are necessarily specific for the processing of phobogenic stimuli. Rather, the processing of phobogenic stimuli represents a highly aversive condition and might be a specific case where personally relevant and salient aversive stimuli are processed even during high perceptual load. Generally, we suggest that activation of the amygdala and visual cortex is due to the interplay between the saliency of stimuli and available cognitive resources. Thus, other threat stimuli might be processed in non-clinical populations as well, given that the saliency and the personal importance of these stimuli are sufficiently high. Future studies should use negative and positive affective control stimuli in order to disentangle the general role of valence and arousal for amygdala responses under high perceptual load.

Furthermore, there was a remarkable reduction of the activation of the amygdala by high load regardless of group and picture category. This is in accordance with previous work (for example, [16]) and provides clear evidence that even in the absence of emotional stimuli the activation in the amygdala is affected by attentional conditions. However, in our study, the differential activation to phobia-related vs neutral pictures was stable across load conditions, indicating a dissociation between a general decrease of the amygdala responsiveness regardless of the specific stimuli and intact relative increased amygdala activation to phobia-related vs neutral stimuli during high load.

Remarkably, there were no effects on task performance in spider-phobic subjects as compared to healthy subjects. However, this finding is in accordance with previous results [8]. Furthermore, impairments in task performance are not consistently observed in subjects with phobias (for example, [7,8,54,55]). Moreover, for the kind of task used in the present study, Bishop *et al. *showed differential brain activation in anxious subjects that was not accompanied by indications of behavioral impairment. Thus, effects on brain activation can be dissociated from those on behavioral measures, at least when assessed through reaction times and errors. Future studies should investigate whether amygdala responses can predict other behavioral measures. Furthermore, it would be interesting to investigate whether automatic amygdala activations can be modified by successful psychotherapy and if these responses are associated with therapeutic success in the short and long term.

## Conclusions

Our results indicate a hyper-responsiveness of the amygdala to disorder-related stimuli in phobic subjects that proved to be independent of attentional load when using a task which induces a high load and which has been shown to prevent amygdala activation to threat in high anxious subjects. This suggests that anxiety disorder patients are characterized by a high level of automaticity of their amygdala responsiveness. Although we did not find an effect of perceptual load on differential amygdala responses, future work might aim to investigate whether a further increase of perceptual load may result in different outcomes as revealed in this study. Thus, also in anxiety patients, the amygdala response to threat might be characterized by a relative instead of an absolute automaticity.

## Competing interests

The authors declare that they have no competing interests.

## Authors' contributions

TS participated in the design and the data analysis of the study and drafted the manuscript. AS and JL carried out the experiments. AS and MML established the experimental procedures. AS performed the data preprocessing and analysis and wrote parts of the manuscript. WM participated in the development and coordination of the study. All authors read and approved the final manuscript.
